# Depression and Cognitive Functioning Among Adults Age 60 and Over: United States, 2011-2014

**DOI:** 10.1093/geroni/igaa057.933

**Published:** 2020-12-16

**Authors:** Ellen Kramarow, Debra Brody

**Affiliations:** National Center for Health Statistics, Hyattsville, Maryland, United States

## Abstract

Previous studies have noted an inverse association between depression and cognitive functioning. The objective of this research is to explore this relationship with data from a nationally representative survey containing validated measures of cognition, depression, and other health conditions. The study population was respondents aged 60 and over who completed the examination component of the 2011-2014 National Health and Nutrition Examination Survey (NHANES) (N=3,472). Cognitive tests included the CERAD word list learning trials, measuring immediate and delayed memory, and the Digit Symbol Substitution test (DSST), measuring attention and processing speed. The presence of depressive symptoms was based on a score of 10 or higher out of 27 from the Patient Health Questionnaire (PHQ-9). Statistical analyses included regression models with low cognitive performance (scoring in the lowest 25th percentile) as the dependent variable. Results from regression models showed that having depressive symptoms significantly increased the odds of scoring in the lowest 25th percentile of both the DSST (OR = 3.4) and the CERAD test (OR = 1.7), controlling for age, sex, and race and Hispanic origin. Adding in a measure of heart disease showed an independent effect of heart disease on low cognitive performance (OR = 1.7 for DSST and OR = 1.3 for CERAD test), while the effect of depression was lessened but still statistically significant. In this study, depression is associated with cognitive functioning, but its effect may be attenuated by the presence of other chronic health conditions.

